# A Tale of Three Misters: The Effect of Water Features on Soundscape Assessments in a Montreal Public Space

**DOI:** 10.3389/fpsyg.2020.570797

**Published:** 2020-11-25

**Authors:** Christopher Trudeau, Daniel Steele, Catherine Guastavino

**Affiliations:** ^1^School of Information Studies, McGill University, Montréal, QC, Canada; ^2^Center for Interdisciplinary Research in Music Media and Technology, McGill University, Montréal, QC, Canada

**Keywords:** urban soundscape, pocket park, restoration, urban design, field experiment, water feature

## Abstract

The acoustic environments of small, central urban parks are often dominated by traffic sounds. Water sounds can be used to mitigate the negative impacts of unwanted sounds through masking. Studies comparing the effects of different water sounds are typically conducted using recordings in laboratory settings where ecological validity is limited. An urban redesign project in Montreal took the innovative approach of trying three sequential temporary designs of a new public square, each of which included a distinct water feature that produced a lightly-audible mist. Here we report on a field experiment evaluating the effect of the water feature in each of the three designs. Respondents (*n* = 274) evaluated their experience with the three different designs using questionnaires including soundscape (SSQP) and restorativeness scales, and perceived loudness. The results indicate a significant interaction effect between the water feature and the design of the space, particularly on ratings of chaotic and loud. While two water feature designs had an overall “positive” effect (i.e., less loud and chaotic) on soundscape assessment, the third water feature design produced the opposite effect. These findings hold even after accounting for ambient temperature. This opportunity to test multiple water features in the same space revealed that water features do not automatically improve soundscape assessments. The visual design, function of the space and environmental conditions should be carefully considered and calls for more field studies. We discuss consequences and considerations for the use of water features in public spaces as well as the implications in terms of ecological validity of soundscape studies.

## 1. Introduction

Water features, as a broad category, have wide ranging uses in urban spaces of all sizes as visual landmarks, as gathering spots and as means to escape heat. They also have a range of uses and impacts on the sound environment, including the masking of unwanted sounds, such as traffic noise (Galbrun and Ali, 2013; Ekman et al., 2015). Yet water features come in a variety of shapes and sizes, with different combinations serving different purposes (Galbrun and Ali, 2013). Moreover, the users' sonic experience in a space depends not only on the sound environment, but also the listening context (Schulte-Fortkamp et al., 2007).

Addressing this relationship between sound environment and experience, a body of work on urban soundscape, defined by the ISO as the “acoustic environment as perceived or experienced and/or understood by a person or people, in context” (International Standards Organization, 2014) has focused on human perception. This ISO definition provides a potential framework to study the sound environments of pocket parks, wherein context “includes the interrelationships between person and activity and place” (International Standards Organization, 2014). Soundscape research considers multidisciplinary and mixed methods approaches in characterizing acoustic environments, with an emphasis on human perception, rather than the physical measurements used in traditional noise control approaches (e.g., decibel levels) (Dubois et al., 2004; Brown, 2010). This translates into a shift from the idea of sound as a pollutant to the potential of using sound as a resource (Schulte-Fortkamp et al., 2007).

An emerging question of interest in soundscape studies is on the use of sound as resource in an environment to provide restoration to its users. Restorative environments enable users to recover from the negative effects of noise exposure, including drained cognitive resources, and to reflect upon daily or life issues (Kaplan, 1995). Originally focused on visual environments, the concept of restoration has been extended to include soundscapes (Payne, 2008). As such, the acoustic environment also affords all of the facets of traditional restorative environments.

This study is conducted in the context of this body of soundscape research that emphasizes the context in which sounds are heard, in particular the context in which water features are deployed in the design of a new space. The aim is to balance the experimental control afforded by laboratory studies, where controlling many conditions is relatively easy, with the ecological validity of *in-situ* studies, where context is inherent in the research design. This study reports on the findings of an *in-situ* soundscape questionnaire deployed in a single public space as it underwent three temporary designs, including a misting water feature in each.

## 2. Literature Review

Large urban parks dominated by greenery have been shown to provide psychological restoration for their users (Jansson and Persson, 2010; Nilsson et al., 2010; Refshauge et al., 2012). Small urban public parks, referred to as “pocket parks” (Nordh and Østby, 2013) are often as busy as the surrounding city. The extent to which pocket parks afford restoration remains understudied. Attention Restoration Theory (ART) suggests that high-quality public spaces can have a positive impact on mental well-being as measured through four components: fascination, being-away, compatibility and extent (Kaplan and Kaplan, 1989). Additionally, there is a possible association between the use of restorative spaces and longer-term (i.e., lingering) attention restoration (e.g., Berto, 2005). This implies that a user's visit to high-quality spaces can have lasting effects on learning and work performance.

A laboratory-based study using visual assessments of pocket parks showed that they have the potential to afford recovery and restoration-related activities (Nordh and Østby, 2013). In particular, the potential for socializing activities was found to be an important element in restoration (Peschardt and Stigsdotter, 2013). To our knowledge, the sonic dimension has only more recently been touched upon in a systematic manner (e.g., Payne and Guastavino, 2018; Steele et al., 2019; Senese et al., 2020). In general, positively-perceived soundscapes are associated with positive effects on well-being (Aletta et al., 2016). Nature sounds appear to effect a faster recovery than other types which could be explained by positive emotions associated with nature (Alvarsson et al., 2010).

In the last decade, a number of soundscape scales have been developed and refined to measure human perceptions of acoustic environments and explore variations on what “sound as resource” could mean in practice (Axelsson et al., 2010; Tarlao et al., 2016; Engel et al., 2018). Axelsson and his team (Axelsson et al., 2010) created and validated the Swedish Soundscape Quality Protocol (SSQP), comprised of eight unidimensional scales (pleasant, unpleasant, eventful, uneventful, calm, monotonous, vibrant, and chaotic). The restorativeness of a sound environment has been operationalized using the Perceived Restorativeness Soundscape Scale (PRSS) (Payne and Guastavino, 2018). Developed from the Perceived Restorativeness Scale (PRS) (Hartig et al., 1997), the PRSS addresses each of the four components of ART in relation to its sound environment rather than the physical place (Payne and Guastavino, 2018).

Laboratory studies show that some water features can improve soundscape ratings of parks in urban areas dominated by road traffic noise (Jeon et al., 2012; Galbrun and Ali, 2013; Skoda et al., 2014; Ekman et al., 2015; Hong et al., 2020a; Senese et al., 2020). Small- and medium-sized features increased ratings of pleasantness (Ekman et al., 2015), peacefulness and tranquility (Galbrun and Ali, 2013), as well as restorativeness ratings (for the fascination and being-away components) (Senese et al., 2020). Moreover, adding desirable sounds, including water features, also reduces perceived loudness, though water features do not always increase pleasantness ratings (De Coensel et al., 2011; Hong et al., 2020a,b).

Due to the difficulty of creating control conditions, *in-situ* studies on the effects of water features on soundscape ratings in pocket parks are rare. To the knowledge of the authors, only two such studies exist. An *in-situ* study of a large fountain in an important Stockholm park found no statistically significant direct effects on soundscape ratings attributable to the fountain (Axelsson et al., 2014). The second study in the courtyard of a German building similarly found no statistically significant results from a small, functioning water feature (Skoda et al., 2014). The same study also evaluated the effect of water sounds over headphones on soundscape ratings in the same courtyard and found significant results. The authors argue that the headphones focused the participants' attention to the water sounds and, in the absence of alternate sound sources, tended toward a central response for each rating. Given the importance of the interrelationship between person, activity and place in soundscape assessment, more *in-situ* studies should be completed to complement lab-based studies on water features in urban parks of all sizes.

Studies have also evaluated the mechanisms through which the water features affect soundscape assessment of urban parks. In particular, two types of masking have been introduced to explain how this might occur: energetic masking and informational masking. In energetic masking, a secondary sound source disrupts the processing of the primary signal in the inner ear (Moore, 2012). Informational masking results when a masking sound that varies unpredictably or that is acoustically very similar to the primary signal produces more masking than would otherwise be expected from energetic masking alone (Moore, 2012). Natural streams and fountains using upward jets were more effective than waterfalls at improving ratings of peacefulness and tranquility, suggesting that energetic masking road traffic noise is not the primary mechanism mediating those particular ratings (Galbrun and Ali, 2013). Unlike waterfalls, smaller water features do not produce the same low-frequency content that is produced by road traffic, and energetic masking is therefore not likely to occur. No known research exists on the *in-situ* effects of lightly audible water features in urban public spaces.

The present study addresses these research gaps by focusing on two research questions:

RQ1—Can small water features that are lightly audible in a outdoor urban public space have an observable effect on soundscape *in-situ* assessments?

RQ2—Can the measured effects of a small mister change if it is deployed in different configurations within the same outdoor urban public space?

## 3. Methods

During the summer of 2018, Montreal's Plateau-Mont-Royal borough embarked on the design of a new pocket park from a previously empty space. The ~900 square meter space is open to public streets on the west, north and east sides, while the south side is bordered by an alleyway and row houses that are inaccessible to vehicular traffic. The street to the north is a busy commercial and transportation artery with a lot of vehicular traffic and many pedestrians. The east and west streets are both residential.

The borough collaborated with designers and facilitators from private firms to determine the needs of the local community through a series of public consultations (see the archived web page for more information^1^). Responding to the community input, three designs were created, highlighting these different needs (i.e., conversation, relaxation, and cultural entertainment). Table 1 provides a full description of the design themes. The three designs used temporary amenities to inform options and democratize the process of creating the final design of the space (slated for 2021).

**Table 1:** Description of the designs and the locations of each mister within the space.

**Design**	**Title**	**Theme**	**# of Misters**	**Mister location**	**Dates**
1	“La place dans la place”	This design promotes gathering and meeting around an elevated platform.	1	In the center of the elevated platform.	31 May–24 June
2	“Une nouvelle promenade”	This design was bisected by a walkway. The northern section, adjacent to the busy road, was a waiting space. The southern section, adjacent to the quieter alleyway, was a garden space designed for relaxation.	2	Connected with the gardens near the south side.	02 July–12 August
3	“Un amphithéatre ouvert sur l'avenue”	This design promotes an animated space, featuring a stage in the south-west corner of the space.	2	Two locations: one in the center of the space and the other in a corner of the noisy-side.	20 August–30 October

*Dates provided are the start and end dates for the specific design*.

Each design included benches, planters, platforms, tables, and chairs in different arrangements. As well, each design featured a lightly-audible water feature that sprayed a fine, upward mist of water (hereafter, these features are referred to as misters). Emerging from small holes in the ground, the thin jets of water were sustained and would reach a height between 1.5 and 2 m, depending on the amount of wind. The misters produced a constant sound that was higher in frequency as compared to the human voice. While the same modular components were used for each iteration of the mister, the placement of these differed between designs. Across all designs, the misters were active during roughly 60% of the data collection sessions, allowing for a quasi-experimental approach featuring six conditions (see Table 4 for a more detailed breakdown). In the context of this paper, Design refers to the collection of modular components, as well as their arrangement. While Design includes the misters, this component is given specific attention as a variable of interest because it was not always on.

In Design 1, the misters were configured as part of a central island where users could sit and view the feature, not unlike a traditional fountain. In Design 2, the misters were located mixed in with planters and vegetation in the quietest section of the park. In Design 3, the misters were located in more open spaces with a less clear function, and when the misters were off these spaces were often used by pedestrians.

### 3.1. Sound Level Measurements

**Figure 1:**
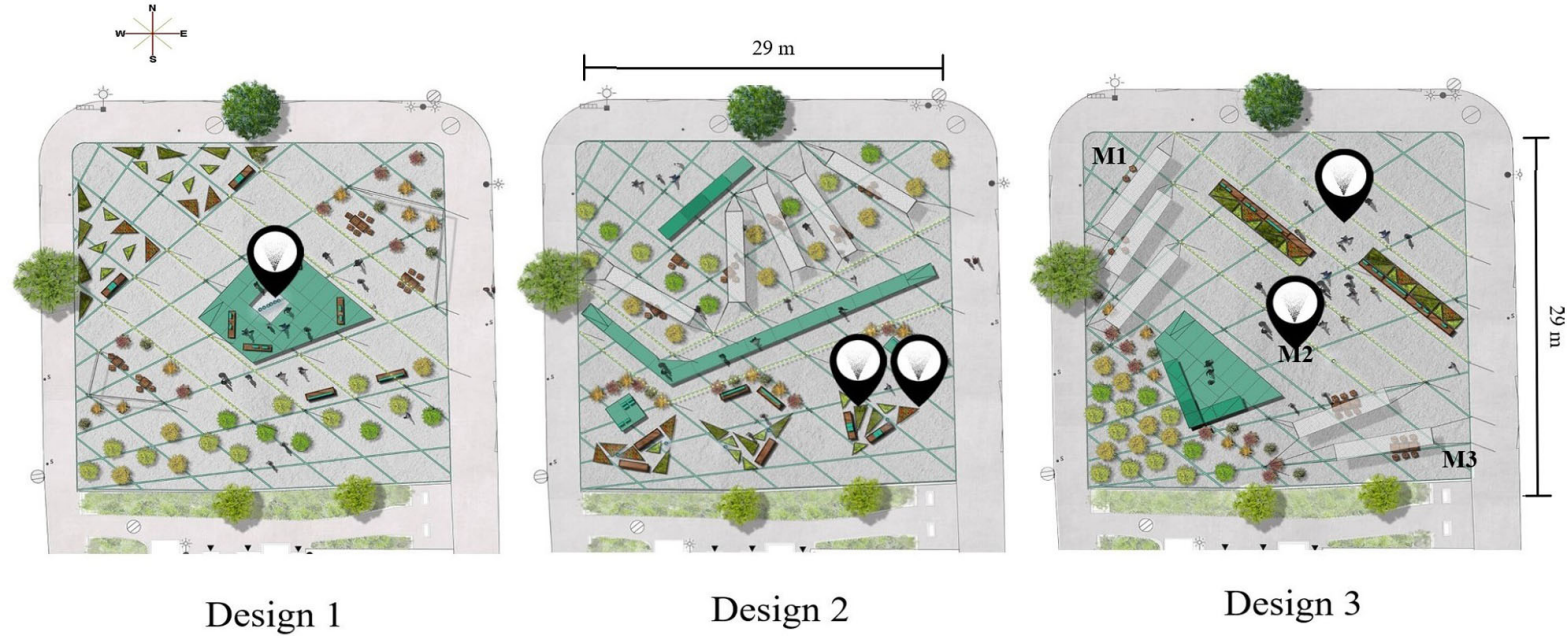
The pins indicate the locations of the misters for each design. In Design 3, there were two misters that were roughly 10 meters apart, one in the center of the space and the other in the northeast corner. The locations M1, M2, and M3 in Design 3 indicate the approximate locations where the sound measurements were made. Design layouts provided by design firm Castor et Pollux and used with permission. Copyright to Cynthia Tarlao for this photo.

Baseline sound level measurements were taken with a B&K 2250 Sound Level Meter, calibrated before use. A total of 24 L_eq, 10min_ measurements were taken when the misters were off. Recordings covered the weekday (Monday and Friday), weekend (Saturday) and evening/night (Friday and Saturday) periods at three different locations in the space (M1—the northwestern corner adjacent to the commercial artery; M2—the center; M3—the southeastern corner adjacent to the residential buildings; see Figure 1). There were no major events (e.g., construction projects, festivals) in the vicinity of the space during questionnaire taking that would strongly influence the sound level. Measurements taken at Location M1 ranged from 61.9 to 66.5 dBA, while those at Locations M2 and M3 were lower (M2: 57.9–61.7 dBA; M3: 57.3–61.4 dBA). These measurements justified the division of the space into the “noisy side,” on the northern half, and the “quiet side,” on the southern half.

### 3.2. Questionnaire Instrument

During each design, users of the space were asked to complete a questionnaire about the soundscape. The questionnaires included scales measured using 5-point Likert-scales that were drawn from the SSQP and PRSS scales. From the SSQP, we included the pleasant, monotonous, vibrant, chaotic, calm and eventful scales. The standard SSQP also includes two additional factors that were not included in our questionnaire: (1) uneventful, as it does not have an adequate translation in French; and (2) unpleasant, as it is so heavily correlated with pleasant (Tarlao et al., 2016). One scale was used from the PRSS: taking a break from the daily routine. In addition, two scales were added to the questionnaire to measure appropriateness for the respondent's activity and the perceived loudness of the space. Table 2 provides more detailed information about the questionnaire construction, including the scales used (the full versions of both the English and French questionnaires can be found in the Supplementary Material). In the interest of clarity, these nine ratings will be collectively referred to as soundscape scales.

**Table 2:** Detailed listing of the questions respondents answered that are relevant to the analysis in this paper.

**Section**	**Question**	**Type**	**Scale**
Activity	*What brings you here today?*	Open-ended	
Sound sources	*Please list below the sounds/noises that you are hearing around you*.	Open-ended	
Soundscape evaluation	I find this soundscape to be:		
	*Pleasant*	Likert-scale	SSQP
	*Appropriate for my activity*	Likert-scale	–
	*Monotonous*	Likert-scale	SSQP
	*Vibrant*	Likert-scale	SSQP
	*Chaotic*	Likert-scale	SSQP
	*Calm*	Likert-scale	SSQP
	*Eventful*	Likert-scale	SSQP
	*Spending time in this soundscape gives me a break from my day-to-day routine*	Likert-scale	PRSS
	*I find the sound level here to be loud*	Likert-scale	–
Demographic information	*I am*	Multiple choice *(Man/Woman/Other)*	
	*I was born in the year*	Open-ended	

*The order of the questions in the table matches that of the questionnaire. The full English and French versions of the questionnaires can be found in the Supplementary Material*.

Respondents were also asked to list the sounds that they heard in the space and provide demographic information at the end of the questionnaire.

### 3.3. Procedure

Questionnaires were collected during 11 sessions, each of which lasted between 1 and 3 h, covering the hours of 11:00 a.m. to 9:00 p.m. The sessions varied in length due to weather, temperature and respondent availability, and were carried out on both weekdays (*n* = 7) and weekends (*n* = 4). Respondents were approached about taking the paper-based questionnaire after having already been in the space for at least 2–3 min. Some of the respondents were alone (*n* = 129) and others were in groups (*n* = 145). Respondents were able to complete the questionnaire using pen and paper in the language of their choice, English or French, and we collapsed the data across languages. Tarlao et al. (2019) provides a description of the differences between French and English responses.

**Figure 2:**
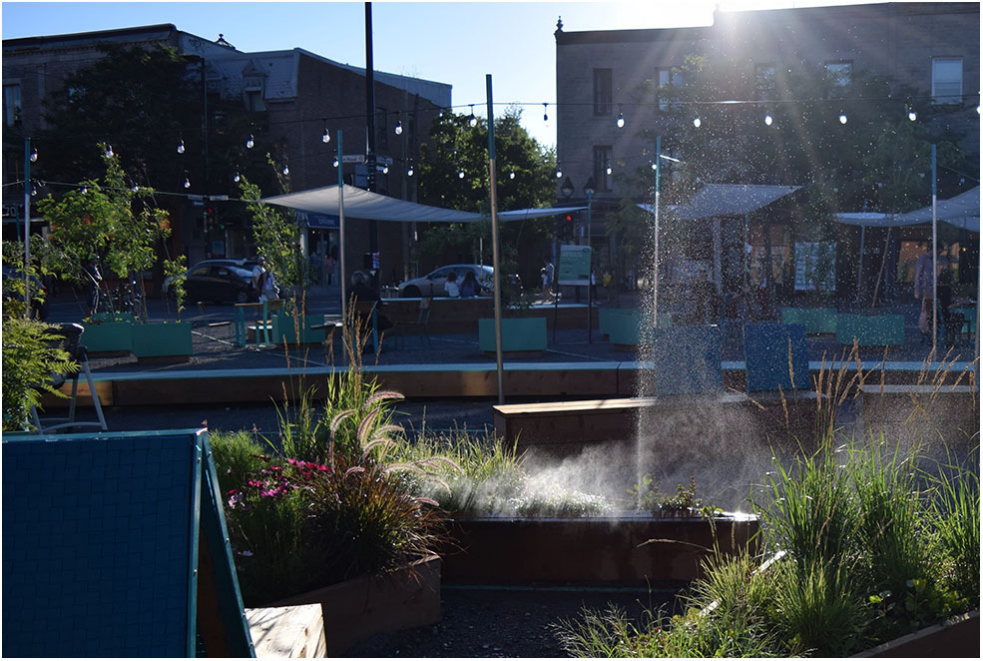
Picture of the mister from Design 2. The effect and jet of the misters for each design was similar. Copyright to Cynthia Tarlao for this photo.

In addition to questionnaire data, we tracked for each respondent the design (1, 2, or 3) and whether the mister was on or off (0 = off; 1 = on). Figure 2 shows the effect and jet of the misters for design 2, though designs 1 and 3 were similar. For designs 2 and 3, we noted the respondents' location within the space. Based on the sound levels of the space and the conceptual designs of the space, these locations were then collapsed into only two areas: a noisy and a quiet side. Respondents were distributed almost evenly between the two halves of the space during the second and third designs. Additionally, Montreal temperature data was scraped from the website for Historical Climate Data from the Government of Canada.^2^ The temperatures ranged between 16.5 and 32.7°C, with a small positive correlation between the temperature and the design (*r* = 0.27, *p* = 0.00007), suggesting a small but significant increase in temperature from Designs 1 to 3.

### 3.4. Open-Ended Question Categorization

The sounds listed by respondents were grouped into one of six categories: water, human, traffic, mechanical, nature, music and other. The categorization was mutually-exclusive, so that each source was only placed in one of the six categories. Table 3 provides the definitions of each category and some examples of sources mentioned by respondents. Explicit mentions of water sounds (e.g., “fountain,” “mister,” “water”) were categorized from this list, and a dummy variable was created to represent whether the respondent mentioned water as a sound source (0 = No, 1 = Yes).

**Table 3:** Sounds mentioned by respondents were categorized into one of six groups.

**Term**	**Definition**	**Examples**
Human	Any source where the sound is the direct result of human activity or speech. Does not include music.	“Peoples' voices,” “skateboards”^3^
Mechanical	Any non-vehicular mechanical source, especially construction and HVAC equipment.	“Supermarket AC”^4^
Music	Any sound created by a musical instrument, whether amplified or not.	“Guitar,” “soft percussion,” “tibetan bong,” “bagpipes”^5^
Nature	Any sound produced by nature. Excludes water-related sources.	“Birds,” “cicada,” “wind”^6^
Traffic	Any sound produced by a part or all of a vehicle propelled by motor.	“Cars,” “traffic,” “planes”^7^
Water	Any sound produced by the movement of water, regardless of the actual source of the sound.	“Mist sound,” “sprinkler,” “the mist”^8^
Other	All remaining responses	“Ambient sounds”^9^

*Definitions are provided in this table, along with examples taken from the questionnaires completed by respondents. Some of the examples are originally in French for which translations are provided here. The originals can be found in the endnotes*.

### 3.5. Profile of the Respondents

In all, there were 274 respondents aged 18–84 (mean of 38 and a standard deviation of 15). Nine respondents did not provide their age. There were more women (*n* = 154) respondents than men (*n* = 111) respondents, though it does not represent a significant imbalance (χ^2^, *df* = 2, *p*-value = 0.19). Nine respondents indicated “other” or “prefer not to answer” as gender. Table 4 shows the demographic breakdown of the respondents per Design.

**Table 4:** Number of questionnaires completed by respondents for each design showing the mister status and gender and age distribution.

**Design**	**Mister**		**Gender**		**Age**			
	**Off**	**On**	**Women**	**Men**	**Min**	**Max**	**Mean**	**Median**
1	25	75	64	32	18	77	39	34
2	15	61	33	39	18	73	35	30
3	70	28	57	40	19	84	38	33
Total	110	164	154	111	–	–	–	–

### 3.6. Statistical Analyses

The Likert-scales were converted to numbers to derive descriptive statistics (Strongly Disagree = 1; Disagree = 2; Neutral = 3; Agree = 4; Strongly Agree = 5). Missing values were replaced with the mean value of that scale, collapsed over all conditions. The number of missing values depended on the scale, but ranged between 1 (0.4%) and 18 (7%).

In order to investigate the effect of the mister and the design on the soundscape scales, we fit a 3 (design) × 2 (mister status) factorial MANCOVA as independent variables and temperature as a covariate. A MANCOVA test extends a standard MANOVA to include a covariate that cannot be accounted for through experimental design, as is the case for temperature in this study. Given the imbalance in the number of respondents for each of the six conditions, we ran a Levene test which was not significant, suggesting non-homogeneity of variance. Thus, we used Pillai's trace for our MANCOVA test to account for the non-homogeneity of variance. Significant MANCOVA results were further investigated using factorial ANCOVA tests for each scale separately. Finally, *post-hoc* tests were performed to determine which conditions account for changes in the scales. All *p*-values were adjusted using the Holm correction. This analysis was performed separately using two binary variables. In the first analysis, the variable was the status of the mister. In the second analysis, the variable was whether the respondent mentioned hearing water sounds (e.g., “water fountain”).

In order to better understand the effect of the misters on the soundscape scales, we performed analyses using the data on respondent location and the sound sources they mentioned. As the mister is only lightly audible, we first investigated the possibility that the Design 2 misters located in the quiet side of the space, did not have the same significant effect on soundscape scales as in the noisy side of the space. To test this hypothesis, we performed ANCOVA tests on the chaotic and loudness scales using the location of the respondent and the status of the misters as independent variables, with temperature as a covariate. Given that only responses from Design 2 were used, Design was not included as an independent variable in these tests.

We also wanted to determine whether the mister had the effect of displacing traffic sounds (e.g., cars, buses). We performed a logistic regression of mentions of traffic sounds, using the design and status of the misters as independent variables. Logistic regression indicates the log odds of the occurrence of a binary outcome, as is the case of the mention (or not) of traffic sounds by questionnaire respondents.

All statistical analyses were performed using R version 3.6.2 (R Core Team, 2020).

## 4. Results

**Figure 3:**
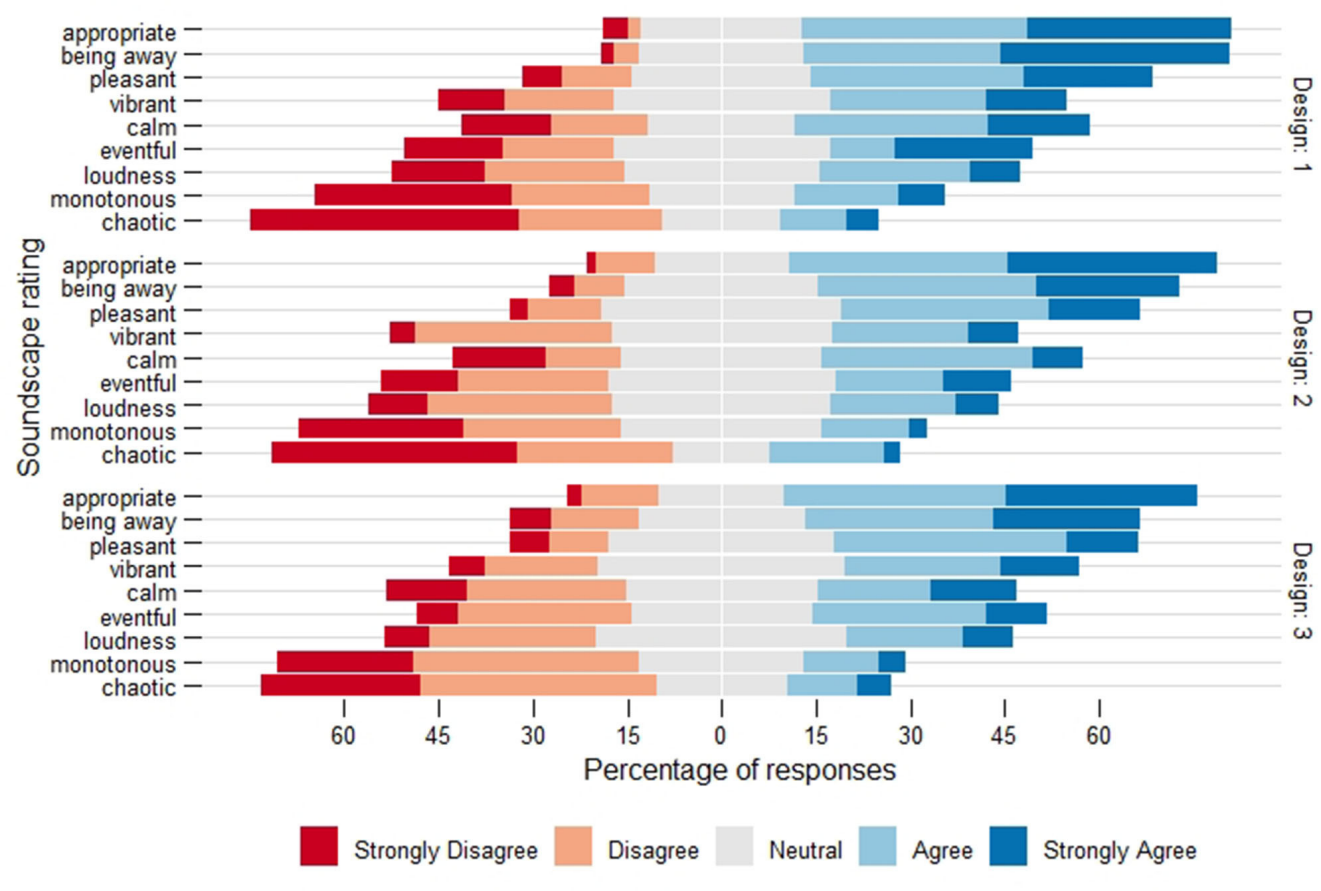
Distribution of the Likert-scale soundscape ratings for each design. Respondents generally found the sound environment was appropriate for activity, provided an opportunity for a break and was pleasant. As well, they disagreed that the sound environment was monotonous and chaotic. No significant differences were found across designs.

In general, respondents rated the sound environments of all three designs as pleasant and appropriate for their activity, and found that these environments provided an opportunity for a break. Regardless of the condition, the average response for all three scales was above the mid-point (i.e., agree/strongly agree). As well, they rated monotonous and chaotic below the mid-point (i.e., disagree/strongly disagree). The respondents were more divided on ratings of vibrant, calm, eventful and loudness. Figure 3 shows the complete distribution of the soundscape ratings along the Likert-scale (i.e., strongly disagree, disagree, neutral, agree, strongly agree) provided by respondents, broken down by design. Moreover, the differences between the soundscape ratings for each design were relatively small (all <0.45 on a 5-point scale), none of which reached statistical significance.

### 4.1. Misting Water Feature

The interaction of the mister and the design had an overall effect on respondents' soundscape evaluations according to the MANCOVA of all scales against the six conditions (see Table 5 for full results). The test also indicated that temperature alone had a significant effect on the soundscape ratings. However, temperature was included as a covariate in the analysis because it could not be controlled through experimental design, and therefore significant results from this variable are not discussed further. No other direct effect was found to be statistically significant, either from the mister or the design. Thus, after controlling for the effect of the daily temperature, the mister had a significant effect on soundscape evaluations that changed depending on the design of the space.

**Table 5:** Results of the MANCOVA test including the status of the water mister.

**Variable**	**Df**	**Pillai**	**Approx F**	**Num Df**	**Den Df**	**Pr (>F)**	
Temperature	1	0.0804	2.517	9	259	0.009	^**^
Design	2	0.061	0.908	18	520	0.569	
Mister	1	0.013	0.373	9	259	0.947	
Design × Mister	2	0.121	1.861	18	520	0.019	^*^
Residuals	267	NA	NA	NA	NA	NA	

*Shows that the interaction effect of design and mister are statistically significant at the p < 0.05 level*.

** *p < 0.01*;

* *p < 0.05*.

Looking at the individual soundscape ratings, we observed a significant interaction effect of mister status and design on the chaotic and loudness scales using a factorial ANCOVA. With the exception of temperature, no other significant effects were found (Table 6 shows the results for the chaotic and loudness scales only).

**Table 6:** Results from the factorial ANCOVA for the chaotic and loudness scales.

**Variable**	**Term**	**DF**	**Sum Sq**.	**Mean Sq**.	***F*-Stat**	**Pr (>F)**	**Adj. Pr (>F)**	
Chaotic	Temperature	1	10.94	10.94	8.70	0.003	0.03	^*^
	Design	2	2.65	1.33	1.05	0.35	1.00	
	Mister	1	0.49	0.49	0.39	0.53	1.00	
	Design × Mister	2	17.18	8.59	6.83	0.001	0.01	^*^
	Residuals	267	335.75	1.26	NA	NA	NA	
Loudness	Temperature	1	2.76	2.76	2.44	0.12	0.72	
	Design	2	1.03	0.514	0.45	0.64	1.00	
	Mister	1	2.60	2.60	2.29	0.13	1.00	
	Design × Mister	2	15.38	7.69	6.79	0.001	0.01	^*^
	Residuals	267	302.16	1.13	NA	NA	NA	

*There was a significant interaction effect for design and mister*.

* p < 0.05.

**Figure 4:**
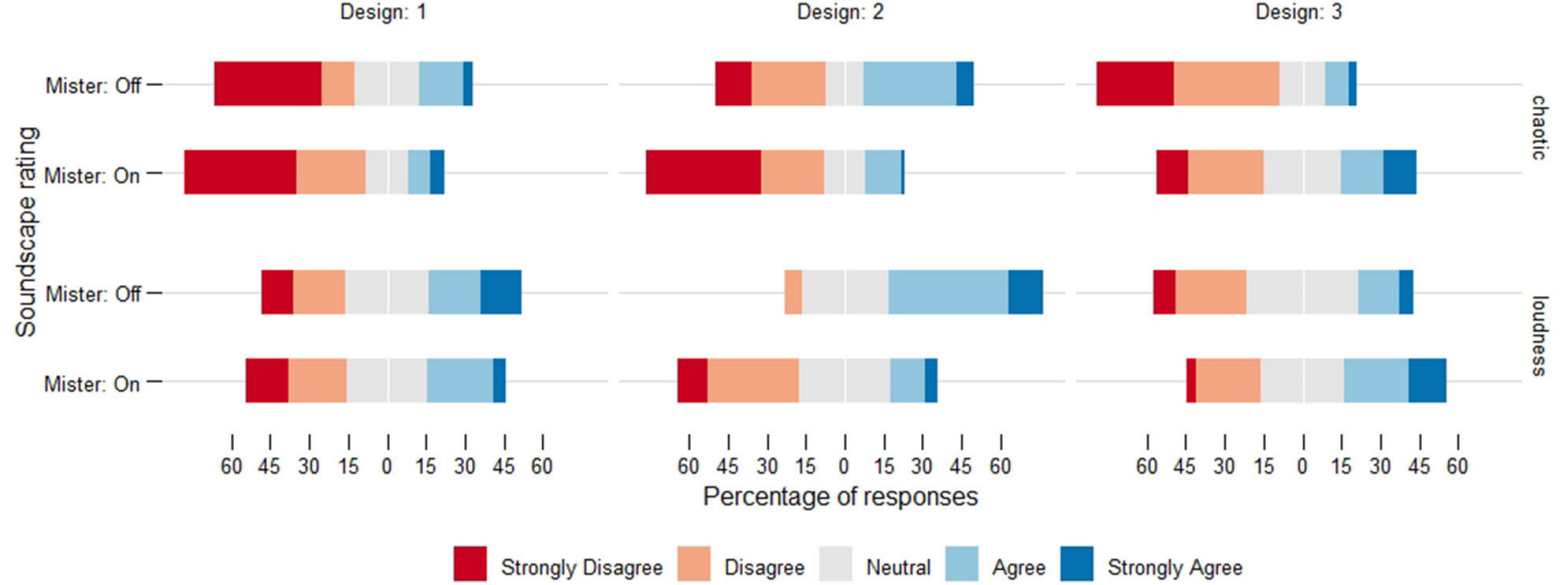
Distribution of the Likert-scale responses for chaotic and loudness only. Each subplot represents a single condition (design and status of the mister).

**Figure 5:**
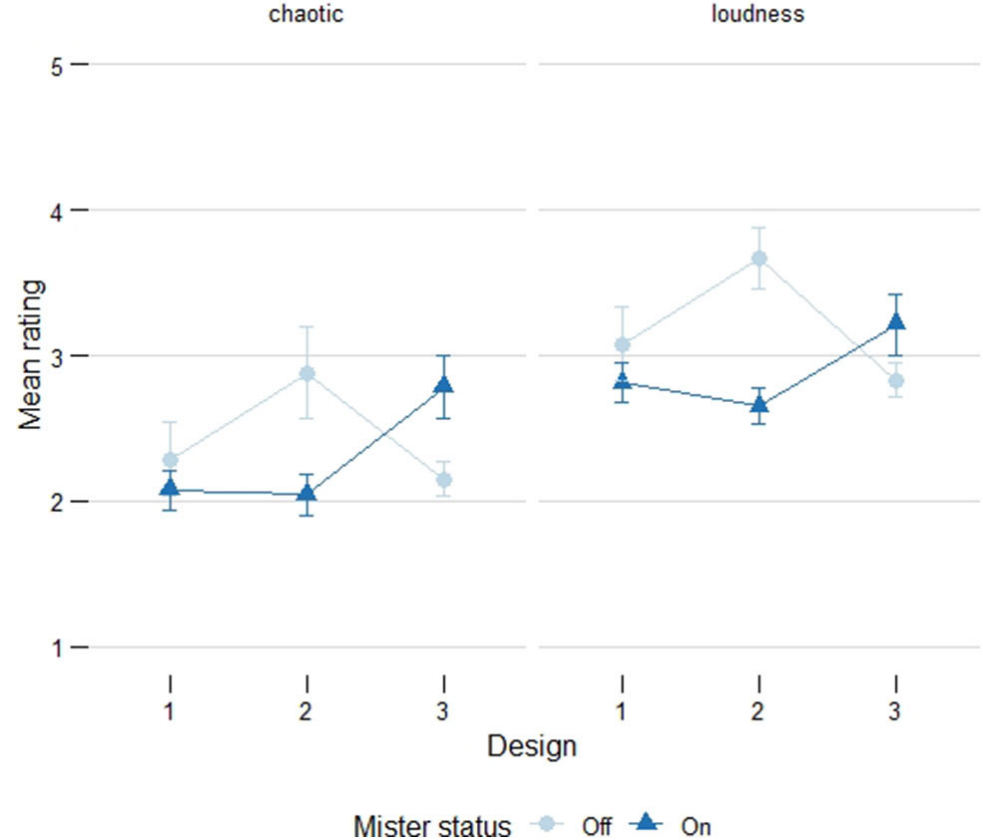
Mean values for the chaotic and loudness ratings. The x-axis indicates the design and the lines compare the status of the mister (off/on). Errorbars indicate the standard error. The mean rating for chaotic and loudness scales was lower when the mister was on during Designs 1 and 2, but they were higher for Design 3, indicating that the mister did not have a consistent effect across designs.

During Designs 1 and 2, the mean values for both chaotic and loudness ratings were lower when the mister was on, with a larger percentage of respondents disagreed with both ratings (i.e., responded disagree/strongly disagree). The exact opposite occurred during Design 3: the mean values for chaotic and loudness were higher when the mister was on and a higher percentage agreed with both ratings (i.e., responded agree/strongly agree) (see Figure 4 for the distribution of responses and Figure 5 for the mean values).

### 4.2. Water as Sound Source

In all, water was mentioned by 66 respondents (representing 40% of all respondents when the fountain was on). All mentions of water sounds occurred when the mister was active. Across the three Designs, respondents mentioned water sounds more frequently during Designs 1 and 2 than in Design 3 (see Table 7). A chi-squared test confirms that this difference is statistically significant (χ^2^ = 13.29, *df* = 2, *p*-value = 0.001).

**Table 7:** Comparison of the mean number of sources mentioned when water is one of them and when it is not (standard deviation in italics).

**Design**	**Count of respondents**		**Mean # of sources** ***(SD)***	
	**Water mentioned**		**Water mentioned**	
	**No**	**Yes**	**No**	**Yes**
1	64	36	3.1 *(1.7)*	3.9 *(1.2)*
2	60	16	3.6 *(1.7)*	4.0 *(1.2)*
3	84	14	3.4 *(1.9)*	4.3 *(1.3)*

*Most respondents did not mention water sounds, but the proportion was higher in Design 3*.

Further analysis of the mentions of different sound sources suggests that the addition of the mister did not remove negative sounds (e.g., traffic sounds) through energetic masking. The average number of sound sources mentioned by respondents increased by 0.8 when one of those sounds was water, as indicated in Table 7, which shows the average number of sounds mentioned for those who did and for those who did not mention water sounds.

Respondents who mentioned water sounds rated the soundscape scales differently than those respondents who did not mention water sounds, even after accounting for temperature. This was confirmed by a 3 (design) × 2 (water mentioned) factorial MANCOVA investigating all nine soundscape scales as dependent variables. This was a main effect and was therefore independent of design of the space (see Table 8 for the complete results of the MANCOVA test).

**Table 8:** Results of the MANCOVA test including a factor for whether water was mentioned by the respondent.

	**Df**	**Pillai**	**Approx F**	**Num Df**	**Den Df**	**Pr (>F)**	
Temperature	1	0.080	2.495	9	259	0.009	^**^
Design	2	0.060	0.900	18	520	0.579	
Water mentioned	1	0.063	1.934	9	259	0.048	^*^
Design × Water mentioned	2	0.089	1.348	18	520	0.153	
Residuals	267	NA	NA	NA	NA	NA	

*When respondents mentioned sources of water sounds, their responses were significantly different*.

** *p < 0.01*;

* *p < 0.05*.

**Figure 6:**
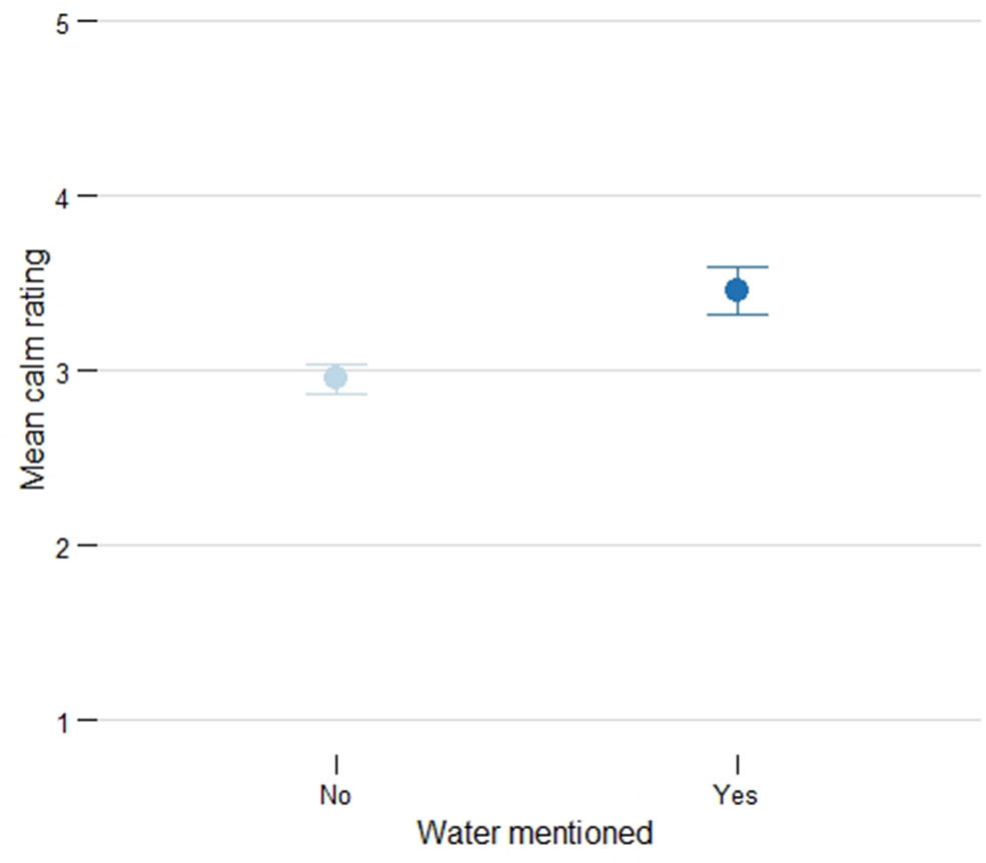
The mean calmness rating increases when the respondent mentions water sounds.

Analysing each of the soundscape scales individually through a factorial ANCOVA test found a marginally significant result for the calmness scale (**df** = 1, *F* = 9.91, *p* = 0.08). Across all designs, the sound environment was rated as calmer when respondents mentioned water sounds than when they did not mention water sounds (see Figure 6 for the mean values).

### 4.3. Effect of Mister as a Function of Location

The misters contribute only a lightly audible misting sound to the space. Given the arrangement of the misters on the quiet side of the space for Design 2, the misting sound was difficult, if not always impossible, to hear on the noisy side. This is confirmed by the proportion of those who mentioned water sounds in the noisy side of the space (1 out of 45 respondents) vs. those in the quiet side of the space (15 out of 31 respondents). Despite only one respondent making a specific mention of water sounds, there was no significant effect of location on the chaotic and loudness ratings, either as main or interaction effects. This was confirmed by the 2 (location: noisy vs. quiet) × 2 (mister status) ANCOVA tests using the chaotic and loudness scales as dependent variables.

### 4.4. Logistic Regression of Traffic Mentions Against Mister

The likelihood of a respondent mentioning traffic sounds was not significantly affected by either the status of the mister or the design of the space according to a logistic regression test. There was also no interaction effect between the two variables (mister status and design). Indeed, across all six conditions (3 design × 2 mister) 86% or more of the respondents indicated hearing traffic sounds.

## 5. Discussion

Despite the heavy volume of vehicular traffic that was audible throughout the space, the sound environment provided an opportunity for restoration to users. Support for this argument can be seen in the extent to which respondents agreed that the sound environment was appropriate for their activity, was pleasant and provided a break across all three Design conditions. The apparent paradox of a potentially restorative space dominated by traffic noise reinforces the importance of considering more than just decibel levels. Indeed, it supports the argument that sound can be a resource as put forward by the soundscape approach (Schulte-Fortkamp et al., 2007). In particular, the sound environment of this urban space was a resource that afforded respondents a break in their daily routine, given that, on average, they rated this scale above the mid-point across all design conditions. This extends other studies that have looked at restoration along the sonic dimension (Payne and Guastavino, 2018; Steele et al., 2019; Senese et al., 2020).

### 5.1. Effectiveness of a Mister in a Small Urban Public Space

RQ1: Can small water features that are lightly audible in a outdoor urban public space have an observable effect on soundscape in-situ assessments?

The mister had no direct significant effect on any of the SSQP scales we used. Though they were studying a larger fountain, Axelsson et al. (2010) similarly found that the water feature did not significantly affect soundscape ratings. This contradicts laboratory-based studies where significant effects were found (Jeon et al., 2012; Galbrun and Ali, 2013; Ekman et al., 2015; Hong et al., 2020a; Senese et al., 2020) and suggests that the context of the urban public space plays an important role in soundscape assessments. The use of headphones and other laboratory equipment could focus the respondent's attention toward the added water feature, making it artificially more effective (Skoda et al., 2014). However, when in the context of an urban public space, the respondent's attention is more scattered, which could reduce the effectiveness of a water feature on soundscape assessments (Skoda et al., 2014). Moreover, as Axelsson et al. (2010) suggest, improving soundscape quality is not as simple as adding a water feature. This appears to also be true for small misting jets.

As well, the mister did not significantly affect the way respondents rated the being-away scale. While this suggests that the mister does not add to the affordance for restoration offered by a sound environment under these conditions, this may be related to the use of a single scale to represent restoration. This contrasts with previous research that reports that water features support psychological restoration along the being-away and fascination scales (Senese et al., 2020). The use of a single scale for restoration in our study is an important limitation that will be addressed in future iterations of our questionnaire. A significant effect may have been found if other ART scales were used to reflect fascination, extent and compatibility. For example, the mister in Design 1 was a focal point for the space that draws in the attention of its users, suggesting that fascination is an appropriate scale to use.

### 5.2. Configuration of the Mister Within the Space

RQ2: Can the measured effects of a small mister change if it is deployed in different configurations within the same outdoor urban public space?

When we consider the effect of the fountain in the context of a specific Design, we note that there is a significant effect. Thus, while a mister on its own is not universally effective at improving soundscape assessments, its integration can be beneficial or it can have the opposite of the intended effect.

We found that the mister had a significant lowering effect on the perceived chaoticness and loudness of the space in Designs 1 and 2. These two findings are in agreement with the laboratory findings cited in the literature review that smaller water features can have desirable effects on soundscape ratings in a pocket park that is dominated by road traffic (Galbrun and Ali, 2013; Ekman et al., 2015). In particular, it aligns with the findings of lower perceived loudness (see De Coensel et al., 2011; Hong et al., 2020a). It is also consistent with findings that upward jets are effective at improving soundscape ratings (Galbrun and Ali, 2013). However, in Design 3, the mister is associated with increased ratings for chaotic and loudness.

### 5.3. Broader Discussion

It is unclear why the mister decreased chaotic and loudness ratings in Designs 1 and 2, while increasing them in Design 3. The literature has often promoted energetic masking as a strategy to deal with unwanted road traffic noise, though this is not possible with smaller water features because they do not generate the necessary low-frequency content (Galbrun and Ali, 2013), and we assume were not sufficiently loud for the task of energetic masking either. We know from the sound sources mentioned by respondents that traffic noise was heard (and named) even when the mister was on, effectively ruling out the possibility that the mister reduced loudness through an energetic masking of traffic noise.

Another possibility is that the sound of the mister is generally considered to be pleasant and desirable and adding such desirable sounds can positively impact soundscape ratings (De Coensel et al., 2011). Moreover, this is consistent with the finding that natural streams and upward pointing jets are preferred over other types of fountains (Galbrun and Ali, 2013). In this scenario, the audible informational content provided by the misters in all 3 designs acts on the perceptions of the users of the space. Informational masking could play a role in reducing chaotic and loudness ratings, though further research is required to validate this possibility. Moreover, this does not account for the change in ratings on the noisy side of the space during the second design where the mister was mostly not audible and was mentioned only once (during a weekend when there is reduced road traffic). While it is possible that respondents heard the mister but did not mention it as a sound source, that is unlikely given how few respondents mentioned the water sounds.

The soundscape approach emphasizes that expectation and perception play an important role in the person's context when they are assessing their sound environment (Dubois et al., 2004; Schulte-Fortkamp et al., 2007). Given that the space is small and that the mister is visible from every angle, it is possible that the mister affected respondents' expectations of the space which, in turn, affected the soundscape ratings. In Designs 1 and 2, the misters were configured in the space so that they would complement other activities (e.g., reading, eating). As such, the presence of the misters attracted people to the space, shifting the emphasis of the soundscape from traffic noise toward human activity. This possible mechanism could also explain why the same misting jets in the same urban space, but with a different configuration, actually increased chaotic and loudness ratings: the water spewed out onto gravel intended for human activity, making the area muddy and unusable. In the context of Design 3, however, the soundscape became less filled with dynamic human activity, and instead more chaotic and traffic-related. As such, this study provides tentative evidence that soundscape ratings are affected by the expectations about the activities that can take place in a space.

The varying number of mister locations within the space is a limitation of this study. There are challenges imposed on *in-situ* research design in spaces where the configuration itself changes. In our case, Design 1 featured a single mister in the center of the space, while Design 2 had two misters roughly 2 m apart and Design 3 had two misters that were 10 m apart. Furthermore, the Design 1 mister was the prominent focal point of the space, which further contrasts with the misters in Designs 2 and 3. Finally, the misters were not off for exactly 50% of each Design condition, introducing a potential source of bias. It is unclear what impact this had on the soundscape assessments made by the respondents, and there is no discernible trend based on either the number of misters or the distance between them.

A further limitation of this study is the difficulty in tracking the activity of each respondent and whether different types of activities would be more suited toward engaging with water features. The questionnaire contained an open-ended question asking respondents “What brings you here today?” The question was received ambiguously by respondents who either stated what they were doing in the space (e.g., “relaxing,” “on my lunch break”) or why they were in the vicinity of the space (e.g., “tourism,” “getting an ice cream”). Thus, we could not fully explore the role activity played in creating a rich context as laid out by the ISO definition of a soundscape, in which person, place, and activity are interrelated (International Standards Organization, 2014). This is an avenue for future research, as more studies are needed on the effect of activity on soundscape assessments, especially in the case of pocket parks. As well, future contributions are necessary to establish a standardized methodology for collecting activity-related data during soundscape evaluations.

That said, this study confirms that questionnaires can be effectively used in a quasi-experimental research design to evaluate the impact of a water feature on soundscape assessments. Through a combination of SSQP (Axelsson et al., 2010), restorativeness (Payne and Guastavino, 2018) and loudness scales, we were able to capture soundscape evaluations that largely agree with existing literature though add nuance (and some further questions) to this growing body of knowledge. Standardized questionnaires should include more scales from the PRSS to represent different components of psychological restoration.

## 6. Conclusion

This study extends the *in-situ* research on the effect of water features to include lightly-audible misters in outdoor, urban environments. Our analysis shows that in some, but not all conditions, adding a mister can enhance the soundscape. In the context of a small urban space located next to a busy street with heavy pedestrian and vehicular traffic, these changes to the scales can be considered as positive and desirable effects. Showing that a small mister can enhance the soundscape is an important finding given that large fountains are often not possible in small pocket parks. First, the size of a large fountain may crowd out other activities. Second, its cost is often prohibitively high.

Further research is required on the ecological validity of lab-based research involving misters and small water features in small public spaces, given the conflicting findings between lab and *in-situ* research. Laboratory settings can cause the participant to focus on the sound environment in a way that is not possible in multimodal environments. Moreover, in a public space, the user is engaged in an activity that contributes to their soundscape assessment. This is not to say that water features have no effect if users of a space cannot hear them. Instead, the misters might provide visual and experiential appeal even without the auditory component, which affects respondents expectations of the space. It does suggest that the mechanism by which misters affect soundscape ratings in context is not straightforward and needs to be better understood.

This study suggests potential design considerations when using a mister in a public space. First, the dimensions of both the mister and the misting water should be chosen so that it attracts users into the space. While it is not possible to rule out the role of masking, designers can use misters to provide sounds that have semantic meaning and positive associations for the users of the space. Second, the mister should be configured within the space in such a way that does not preclude the likely activities. Therefore, it is important to have a good sense of what users want from the space. The design of the space and the configuration of the mister within it together should clearly indicate to the user the activities afforded by the space. These considerations inform the context that is highlighted by the soundscape approach (i.e., the relationship between person, place and activity) and in turn can be used to improve the soundscape of a small public space.

## Data Availability Statement

The datasets generated for this study will not be made publicly available. Requests to access these datasets should be directed to the corresponding author.

## Ethics Statement

The studies involving human participants were reviewed and approved by McGill's Research Ethics Board (REB-2), McGill University (REB-55-0615). Written informed consent for participation was not required for this study in accordance with the national legislation and the institutional requirements.

## Author Contributions

CT and DS collected and analyzed the data under the guidance of CG. All authors designed the experiment and contributed to the writing of the manuscript.

## Conflict of Interest

The authors declare that the research was conducted in the absence of any commercial or financial relationships that could be construed as a potential conflict of interest.

## Footnotes

^1^https://web.archive.org/web/20190301152402mp/https://www.realisonsmtl.ca/962mont-royal

^2^https://climate.weather.gc.ca/

^3^“voix des personnes,” “skateboards.”

^4^“Air climatisé du supermarché.”

^5^“Guitare,” “percussions douces,” “bong tibetain,” “cornemuses.”

^6^“Oiseaux,” “cigale,” “vent.”

^7^“Avions.”

^8^“La bruine.”

^9^“Bruits ambiants.”
