# Supplementary material for: A Tale of Three Misters: The Effect of Water Features on Soundscape Assessments in a Montreal Public Space
Source: Front Psychol. 2020 Nov 25;11:570797. doi: 10.3389/fpsyg.2020.570797 (PMC7723869; doi:10.3389/fpsyg.2020.570797)
Supplement: Supplementary file 1 [file Data_Sheet_1.PDF]

Please answer each question to the best of your ability. There is no right or wrong answer.

What brings you here today?	
How would you describe the present ambiance of this space?	

Please list below the sounds/noises that you are hearing around you into the column that applies.

Pleasant	Unpleasant	Neutral
• • •	• • •	• • •

The ***soundscape*** *is the collection of all the sounds and noises that you hear around you.*

For each question below, circle one response:

	Strongly disagree				Strongly agree
I find this **soundscape** to be:					
Pleasant	1	2	3	4	5
Appropriate for my activity	1	2	3	4	5
Monotonous	1	2	3	4	5
Vibrant	1	2	3	4	5
Chaotic	1	2	3	4	5
Calm	1	2	3	4	5
Eventful	1	2	3	4	5
Spending time in this soundscape gives me a break from my day-to-day routine	1	2	3	4	5
I find the sound level here to be loud	1	2	3	4	5
In general, I am sensitive to noise	1	2	3	4	5
I see myself as extraverted, enthusiastic (that is, sociable, assertive, talkative, active, NOT reserved or shy)	1	2	3	4	5

I am:	a man	a woman	other/prefer not to say	
Today, I am here:	alone	with others (2 to 4 people)	in a group (5 or more)	
I visit this space:	for the first time	at least once a month	at least once a week	
I live:	a block or two from here	elsewhere in the Plateau	elsewhere in Montreal	outside of Montreal

In this space, I would like to hear:	
Comments:	

I was born in the year: ______

Date and time: MM / DD 2018 : AM/PM

McGill

Thank you for your participation.
