# Supplementary material for: A Tale of Three Misters: The Effect of Water Features on Soundscape Assessments in a Montreal Public Space
Source: Front Psychol. 2020 Nov 25;11:570797. doi: 10.3389/fpsyg.2020.570797 (PMC7723869; doi:10.3389/fpsyg.2020.570797)
Supplement: Supplementary file 2 [file Data_Sheet_2.PDF]

Veuillez répondre à chaque question du mieux que vous pouvez. Il n'y a pas de bonne ou de mauvaise réponse.

Qu'est-ce qui vous amène ici aujourd'hui ?	
Comment décririez-vous l'ambiance de ce lieu en ce moment?	

Listez ci-dessous les sons et bruits que vous entendez dans ce lieu en ce moment.

Agréables	Désagréables	Neutres
•<br>•<br>•	•<br>•<br>•	•<br>•<br>•

<u>L'ambiance sonore</u> *<u>désigne l'ensemble des bruits et sons que vous entendez autour de vous.</u>*

Pour chaque question ci-dessous, entourez une réponse :

	Pas du tout d'accord				Tout à fait d'accord
Je trouve l'ambiance ***sonore*** en ce lieu :					
Agréable	1	2	3	4	5
Appropriée pour mon activité	1	2	3	4	5
Monotone	1	2	3	4	5
Dynamique	1	2	3	4	5
Chaotique	1	2	3	4	5
Calme	1	2	3	4	5
Animée	1	2	3	4	5
Passer du temps dans cet environnement sonore me permet de faire une pause dans ma routine quotidienne	1	2	3	4	5
Je trouve le niveau sonore élevé en ce lieu	1	2	3	4	5
Je suis sensible au bruit en général	1	2	3	4	5
Je me considère extraverti(e), c'est-à-dire sociable/ affirmé(e)/ loquace, et non pas réservé(e)/ timide	1	2	3	4	5

Je suis :	Un homme		Une femme	Autre/Préfère ne pas dire
Je suis :	Seul(e)		Accompagné(e) (2 à 4 pers.)	En groupe (5 pers. ou plus)
Je fréquente cet espace :	Pour la première fois		Au moins une fois par mois	Au moins une fois par semaine
Je vis :	À quelques pas d'ici	Ailleurs dans le Plateau	Ailleurs à Montréal	En-dehors de Montréal

Dans cet espace, j'aimerais entendre :	
Commentaires	

Mon année de naissance : ______ Date et heure : Le 2018 à h

McGill

Merci de votre participation.
